# Supplementary material for: Clinical Impact of Functional CYP2C19 and CYP2D6 Gene Variants on Treatment with Antidepressants in Young People with Depression: A Danish Cohort Study
Source: Pharmaceuticals (Basel). 2022 Jul 14;15(7):870. doi: 10.3390/ph15070870 (PMC9318115; doi:10.3390/ph15070870)
Supplement: Supplementary file 1 [file pharmaceuticals-15-00870-s001.zip › pharmaceuticals-1796163-supplementary.pdf]

pharmaceuticals

MDPI

Supplement

Table S1. shows all available single nucleotide polymorphisms (SNPs)

**Gene**	**RS-number**	**Allele nomencla-ture**	**Genomic position**
CYP2C19	rs4244285	*2	10.10:g.96541616G>A
CYP2C19	rs41291556	*8	10.10:g.96535173T>C
CYP2C19	rs12248560	*17	10.10:g.96521657C>T
CYP2D6	rs3892097	*4	22.10:g.42524947C>T
CYP2D6	rs1065852	*4, *10, *14A	22.10:g.42526694G>A
CYP2D6	rs28371706	*17	22.10:g.42525772G>A
CYP2D6	rs28371725	*41	22.10:g.42523805C>T

This table is a shortened version of Lunenburg et al.´s supplement table 3.

Table S2. Genotype-phenotype translation of available variants

Gene	***Predicted diplotype***	***Phenotype***
CYP2C19	*17/*17	UM
CYP2C19	*1/*17	RM
CYP2C19	*1/*1	NM
CYP2C19	*1/*2, *1/*8, *2/*17	IM
CYP2C19	*2/*2,*2/*8, *8/*8	PM
CYP2D6	*1/*1	GAS 2 (normal metabolic capacity)
CYP2D6	*1/*17, *1/*41	GAS 1.5
CYP2D6	*1/*10	GAS 1.25
CYP2D6	*1/*4, *17/*17, *17/*41, *41/*41	GAS 1
CYP2D6	*10/*17, *10/*41	GAS 0.75
CYP2D6	*4/*17, *4/*41, *10/*10	GAS 0.5
CYP2D6	*4/*10	GAS 0.25
CYP2D6	*4/*4	GAS 0
GAS of two alleles for CYP2D6:<br>>2.25 UM<br>1.25 - 2.25 NM<br>>0 - <1.25 IM<br>0 PM		

Abbreviations: UM: ultrarapid metabolizer, RM: rapid metabolizer, NM: normal metabolizer, IM: intermediate metabolizer, PM: poor metabolizer, GAS: gene activity score

This table is a shortened version of Lunenburg et al.´s supplement table 2.

Table S3. Detail description of the covariates and all other variables

Variable	Description/assessing time period
Covariates adjusted in the association analyses	
Age*	Age at index date
Sex**	Sex at birth, Male/Female
Danish region**	Danish region where index prescription was redeemed; categorized as Capital Region of Denmark vs other (Central Denmark Region, North Denmark Region, Region Zealand, & Region of Southern Denmark)
SES (socio-economic status)**	Parents' SES for individuals with age <18, and individuals' own SES for rest of others at index; categorized as employed, on social benefits, on study & others
Number of previous psychiatric diagnoses*	Total number of psychiatric diagnoses any time before index date; diagnoses were counted in a 10 scale of ICD10 F0 to F9
Calendar year *	Calendar year of index prescription
Hospital contacts within the previous year of index date	Number of psychiatric hospital/department contacts within the previous year of index date
Strong CYP2D6 inhibitors**	Using any of the following drugs within the last 90 days before the index date (ATC-code): Bupropion (N06AX12), Paroxetine (N06AB05), Cinacalcet (H05BX01)
Moderate CYP2D6 inhibitors**	Using any of the following drugs within the last 90 days before the index date (ATC-code): Abiraterone (L02BX03), Duloxetine (N06AX21), Mirabegron (G04BD12), Terbinafine (D01BA02)
Weak CYP2D6 inhibitors**	Using any of the following drugs within the last 90 days before the index date (ATC-code): Amiodarone (C01BD01), Celecoxib (C08CA51, L01XX22, M01AH01), Clobazam (N05BA09)<br>Cobicistat (J05AR15, V03AX03, J05AR14, J05AR22, J05AR18, J05AR09), Fluvoxamine (N06AB08), Labetalol (C07AG01, C07CG01, C07BG01), Ritonavir (J05AR23, J05AR26, J05AP52, J05AR10, J05AP53, J05AE03), Vemurafenib (L01XE15)
Strong CYP2C19 inhibitors**	Using any of the following drugs within the last 90 days before the index date (ATC-code): Fluconazole (J02AC01), Fluvoxamine (N06AB08)
Moderate CYP2C19 inhibitors**	Using any of the following drugs within the last 90 days before the index date (ATC-code): Enzalutamide (L02BB04), Voriconazole (J02AC03)
Weak CYP2C19 inhibitors**	Using any of the following drugs within the last 90 days before the index date (ATC-code): Esomeprazole (A02BC05), Modafinil (N06BA07), Omeprazole (A02BC01), Pantoprazole (A02BC02)

CYP2C19 inducers**	Using any of the following drugs within the last 90 days before the index date (ATC-code): Apalutamide (L02BB05), Aprepitante (A04AD12), Carbamazepine (N03AF01), Efavirenz (J05AG03), Enzalutamide (L02BB04), Letermovir (J05AX18), Prednisone (H02AB07), Rifampicin/Rifampin (J04AB02), Ritonavir (J05AE03)
Within last 90ds: Antiepileptic drug use**	Using any antiepileptic drug (ATC-code: N03A) within the last 90 days before the index date.
Within last year: suicide attempt/self-harm	Previous suicide attempt/self-harm within the last year before index date. Algorithm based on ICD and hospital contact reason code 4 (1).
Past ever suicide attempt/self-harm	Past ever history of suicide/self-harm before the index date. Algorithm based on ICD and hospital contact reason code 4 (1).
Additional variables shown in the baseline characteristics table	
Age (in years)**	Age at index as a continuous variable and a group variable (0-18 years, 19-25 years, & 26-36 years)
First prescriber hospital**	Defines if the first prescription was prescribed at a hospital or primary care.
Selective psychiatric diagnoses at index**	At index date if they had an ADHD, schizophrenia, bipolar disorder, affective disorder, depression, or autism diagnosis (Yes/no)
Ethnicity**	Based on parental place of birth. Europe means other countries than Denmark/Scandinavia and Scandinavia means only Norway, Sweden, Finland, and Iceland. If one parent was born outside of Denmark, that region was used. If both parents were born in different regions outside of Denmark, mixed was used.
Family type**	Family type at index year, categorized as single or non-single (living with someone)
Past ever: Selective psychiatric diagnoses**	If they had a specific psychiatric diagnosis any time before index date; list of diagnoses: DF0: Mental disorder due to known physiological conditions, DF1: Substance abuse disorders, DF2: Schizophrenia/schizotypal/delusional disorders, DF3: Mood disorders, DF4: Anxiety/stress-related disorders, anxiety disorders (F40-41,44-48), DF42: OCD, DF43: PTSD, DF5: Behavioral syndromes associated with physiological disorder, DF6: Disorders of adults personality & behavior, DF7: Intellectual disabilities, DF8: Pervasive & specific development disorders, DF9: Behavioral & emotional disorders with onset occurring in child/adolescence, Tic disorders (F95.0-2, F95.8-9)
Past ever: Selective somatic diagnoses**	If they had a specific somatic diagnosis (associated with depression) any time before index date; list of diagnoses (ICD-10 code): Esophageal reflux (K21), diabetes Mellitus type 2 (E110-119), diabetes Mellitus type 1 (E100-109), epilepsy (G400-409), cardiovascular disease (I10-15, I20-25, I60-96, I70-79), asthma (J450-459), rheumatoid arthritis (M050-069), thyroid disease (E050-059, E060-069, E030-039), obesity (E660, E668), migraine (G430-431)

Parents: Psychiatric diagnosis**	If the individuals' parents had a history of past ever psychiatric diagnosis before index date; list of diagnoses (ICD-10 code): Schizophrenia (F20), bipolar disorder (F30-31), depression (F32-33), other affective disorders (F34-39)

*Continuous; **Categorical variables

Table S4. Baseline characteristics of genotyped (included) vs. not genotyped (excluded) individuals born between 1981 and 2005, with a depression diagnosis any time between 1996 and 2012, at index of first time prescription of escitalopram, citalopram, sertraline or fluoxetine.

	Overall, N=20343		Excluded, N=3046		Included, N=17297	
	N	(%)	N	(%)	N	(%)
**Sex**						
Female	14380	(70.7)	2160	(70.9)	12220	(70.6)
Male	5963	(29.3)	886	(29.1)	5077	(29.4)
**Age in groups**						
≤18 years	9059	(44.5)	1169	(38.4)	7890	(45.6)
19-25 years	9117	(44.8)	1456	(47.8)	7661	(44.3)
26+ years	2167	(10.7)	421	(13.8)	1746	(10.1)
**Mean age in years (SD)**	19.9 (3.9)		20.5 (3.9)		19.7 (3.9)	
**First prescriber: hospital**						
Yes	1048	(5.2)	95	(3.1)	953	(5.5)
**Region at index prescription**						
Capital Region	5486	(27.0)	821	(27.0)	4665	(27.0)
Middle Jutland	4870	(23.9)	681	(22.4)	4189	(24.2)
North Jutland	2262	(11.1)	426	(14.0)	1836	(10.6)
Southern Denmark	4202	(20.7)	568	(18.6)	3634	(21.0)
Zealand	3523	(17.3)	550	(18.1)	2973	(17.2)
**Ethnicity#**						
Danish/European	18368	(90.3)	2763	(90.7)	15605	(90.2)
Mixed	1834	(9.0)	264	(8.7)	1570	(9.1)

	Overall, N=20343		Excluded, N=3046		Included, N=17297	
	N	(%)	N	(%)	N	(%)
Outside of Europe	141	(0.7)	19	(0.6)	122	(0.7)
**Family type**						
Missing	195	(1.0)	32	(1.1)	163	(0.9)
Non-single	10168	(50.0)	1432	(47.0)	8736	(50.5)
Single	9980	(49.1)	1582	(51.9)	8398	(48.6)
**Parents/adults SES***						
Missing	388	(1.9)	62	(2.0)	326	(1.9)
Employed	9612	(47.2)	1300	(42.7)	8312	(48.1)
On social benefits	4495	(22.1)	818	(26.9)	3677	(21.3)
On study	4320	(21.2)	645	(21.2)	3675	(21.2)
Others	1528	(7.5)	221	(7.3)	1307	(7.6)
**Within last year: No. of psychiatric hospital contacts**						
0	10617	(52.2)	1534	(50.4)	9083	(52.5)
1	3331	(16.4)	488	(16.0)	2843	(16.4)
2	1313	(6.5)	221	(7.3)	1092	(6.3)
3	630	(3.1)	126	(4.1)	504	(2.9)
4	364	(1.8)	79	(2.6)	285	(1.6)
>4	4088	(20.1)	598	(19.6)	3490	(20.2)
**iPSYCH: ADHD diagnosis at index**						
Yes	562	(2.8)	61	(2.0)	501	(2.9)
**iPSYCH: Schizophrenia diagnosis at index**						
Yes	293	(1.4)	181	(5.9)	112	(0.6)
**iPSYCH: Bipolar diagnosis at index**						
Yes	126	(0.6)	45	(1.5)	81	(0.5)

	Overall, N=20343		Excluded, N=3046		Included, N=17297	
	N	(%)	N	(%)	N	(%)
**iPSYCH: Affective diagnosis at index**						
Yes	8944	(44.0)	1319	(43.3)	7625	(44.1)
**iPSYCH: Depression diagnosis at index**						
Yes	8854	(43.5)	1293	(42.4)	7561	(43.7)
**iPSYCH: Autism diagnosis at index**						
Yes	607	(3.0)	69	(2.3)	538	(3.1)
**Year as category of first prescription**						
1995-2001	615	(3.0)	149	(4.9)	466	(2.7)
2001-2005	5069	(24.9)	1092	(35.9)	3977	(23.0)
2006-2010	10504	(51.6)	1408	(46.2)	9096	(52.6)
2011-2016	4155	(20.4)	397	(13.0)	3758	(21.7)

#Ethnicity was defined based on parental place of birth. Europe means all other countries in Europe except Denmark. If one parent was born outside of Denmark, that region was used. If both parents were born in different regions outside of Denmark, mixed was used.

*For those who had missing information on their own socioeconomic status (SES) we extracted SES from their parents

For a detailed description of all the variables see supplement table 3.

Table S5. Baseline characteristics at the index date of the first time prescription of sertraline, citalopram, escitalopram, or fluoxetine of all included individuals born between 1981 and 2005, with a depression diagnosis any time between 1996 and 2012, age groups (<18, 19-25, and 26-36 years).

	**Age groups**					
	≤18 years, N=7890		19-25 years, N=7661		26+ years, N=1746	
	N	(%)	N	(%)	N	(%)
**Antidepressants**						
Sertraline	2513	(31.9)	1567	(20.5)	503	(28.8)
Citalopram	3111	(39.4)	4263	(55.6)	907	(51.9)
Escitalopram	928	(11.8)	1428	(18.6)	276	(15.8)
Fluoxetine	1338	(17.0)	403	(5.3)	60	(3.4)
**Sex**						
Female	5907	(74.9)	5278	(68.9)	1035	(59.3)
Male	1983	(25.1)	2383	(31.1)	711	(40.7)
**First prescriber: hospital**						
No	7305	(92.6)	7404	(96.6)	1635	(93.6)
Yes	585	(7.4)	257	(3.4)	111	(6.4)
**Region at index prescription**						
Capital Region	2045	(25.9)	2051	(26.8)	569	(32.6)
Middle Jutland	1718	(21.8)	2022	(26.4)	449	(25.7)
North Jutland	958	(12.1)	767	(10.0)	111	(6.4)
Southern Denmark	1595	(20.2)	1632	(21.3)	407	(23.3)
Zealand	1574	(19.9)	1189	(15.5)	210	(12.0)
**Ethnicity#**						
Danish/European	7079	(89.7)	6929	(90.4)	1597	(91.5)
Mixed	754	(9.6)	678	(8.9)	138	(7.9)
Outside of Europe	57	(0.7)	54	(0.7)	11	(0.6)
**Family type**						
Missing	64	(0.8)	74	(1.0)	25	(1.4)
Non-single	4733	(60.0)	3268	(42.7)	735	(42.1)

	Age groups					
	≤18 years, N=7890		19-25 years, N=7661		26+ years, N=1746	
	N	(%)	N	(%)	N	(%)
Single	3093	(39.2)	4319	(56.4)	986	(56.5)
**Parents/adults SES***						
Missing	271	(3.4)	45	(0.6)	10	(0.6)
Employed	4788	(60.7)	2682	(35.0)	842	(48.2)
On social benefits	1360	(17.2)	1749	(22.8)	568	(32.5)
On study	1048	(13.3)	2353	(30.7)	274	(15.7)
Others	423	(5.4)	832	(10.9)	52	(3.0)
**Within last year: No. of psychiatric hospital contacts**						
0	3598	(45.6)	4471	(58.4)	1014	(58.1)
1	1461	(18.5)	1138	(14.9)	244	(14.0)
2	601	(7.6)	414	(5.4)	77	(4.4)
3	261	(3.3)	205	(2.7)	38	(2.2)
4	161	(2.0)	101	(1.3)	23	(1.3)
>4	1808	(22.9)	1332	(17.4)	350	(20.0)
**iPSYCH: ADHD diagnosis at index**						
Yes	268	(3.4)	186	(2.4)	47	(2.7)
**iPSYCH: Schizophrenia diagnosis at index**						
Yes	45	(0.6)	48	(0.6)	19	(1.1)
**iPSYCH: Bipolar diagnosis at index**						
Yes	27	(0.3)	39	(0.5)	15	(0.9)
**iPSYCH: Affective diagnosis at index**						
Yes	3821	(48.4)	2912	(38.0)	892	(51.1)
**iPSYCH: Depression diagnosis at index**						
Yes	3791	(48.0)	2887	(37.7)	883	(50.6)

	Age groups					
	≤18 years, N=7890		19-25 years, N=7661		26+ years, N=1746	
	N	(%)	N	(%)	N	(%)
**iPSYCH: Autism diagnosis at index**						
Yes	430	(5.4)	88	(1.1)	20	(1.1)
**Past: No. of past mental diagnoses**						
0	1797	(22.8)	2484	(32.4)	467	(26.7)
1	2443	(31.0)	2242	(29.3)	489	(28.0)
2	2010	(25.5)	1560	(20.4)	400	(22.9)
3	1029	(13.0)	860	(11.2)	256	(14.7)
4	424	(5.4)	349	(4.6)	85	(4.9)
>4	187	(2.4)	166	(2.2)	49	(2.8)
**Past ever: DF0: Mental disorder due to known physiological conditions**						
Yes	37	(0.5)	53	(0.7)	19	(1.1)
**Past ever: DF1: Substance abuse disorders**						
Yes	573	(7.3)	1061	(13.8)	323	(18.5)
**Past ever: DF2: Schizophrenia/schizotypal/delusional disorders**						
Yes	396	(5.0)	295	(3.9)	89	(5.1)
**Past ever: DF3: Mood disorders**						
Yes	4813	(61.0)	3862	(50.4)	1038	(59.5)
**Past ever: DF4: Anxiety/stress-related disorders**						
Yes	2474	(31.4)	2255	(29.4)	585	(33.5)
**Past ever: DF5: Behavioral syndromes associated with psychological dis.**						
Yes	829	(10.5)	451	(5.9)	93	(5.3)

	**Age groups**					
	≤18 years, N=7890		19-25 years, N=7661		26+ years, N=1746	
	N	(%)	N	(%)	N	(%)
**Past ever: DF6: Disorders of adults' personality & behavior**						
Yes	801	(10.2)	1042	(13.6)	257	(14.7)
**Past ever: DF7: Intellectual disabilities**						
Yes	146	(1.9)	91	(1.2)	19	(1.1)
**Past ever: DF8: Pervasive & specific development disorders**						
Yes	791	(10.0)	231	(3.0)	41	(2.3)
**Past ever: DF9: Behavioral & emotional disorders with onset occurring in childhood/adolescence**						
Yes	1373	(17.4)	877	(11.4)	191	(10.9)
**Past ever: Anxiety disorders (F40-41,44-48)**						
Yes	457	(5.8)	515	(6.7)	137	(7.8)
**Past ever: DF42: OCD**						
Yes	195	(2.5)	119	(1.6)	28	(1.6)
**Past ever: DF43: PTSD**						
Yes	195	(2.5)	119	(1.6)	28	(1.6)
**Esophageal reflux**						
Yes	71	(0.9)	77	(1.0)	15	(0.9)
**Diabetes Mellitus type 2**						
Yes	15	(0.2)	24	(0.3)	14	(0.8)
**Diabetes Mellitus type 1**						
Yes	53	(0.7)	66	(0.9)	18	(1.0)
**Epilepsy**						
Yes	234	(3.0)	214	(2.8)	51	(2.9)

	Age groups					
	≤18 years, N=7890		19-25 years, N=7661		26+ years, N=1746	
	N	(%)	N	(%)	N	(%)
**Cardiovascular disorders**						
Yes	118	(1.5)	219	(2.9)	75	(4.3)
**Asthma**						
Yes	644	(8.2)	485	(6.3)	103	(5.9)
**Rheumatoid arthritis**						
Yes	17	(0.2)	13	(0.2)	5	(0.3)
**Thyroid disease**						
Yes	29	(0.4)	62	(0.8)	15	(0.9)
**Obesity**						
Yes	95	(1.2)	170	(2.2)	105	(6.0)
**Migraine**						
Yes	59	(0.7)	68	(0.9)	25	(1.4)
**Past ever: self-harm/suicide attempt**						
Yes	1350	(17.1)	1145	(14.9)	220	(12.6)
**Within last year: history of self-harm/suicide attempt**						
Yes	807	(10.2)	477	(6.2)	51	(2.9)
**Parents: Had any mental diagnosis**						
Yes	1837	(23.3)	1517	(19.8)	354	(20.3)
**Parents: Schizophrenia**						
Yes	61	(0.8)	39	(0.5)	11	(0.6)
**Parents: Bipolar disorder**						
Yes	103	(1.3)	95	(1.2)	23	(1.3)
**Parents: Depression**						
Yes	757	(9.6)	591	(7.7)	119	(6.8)

	Age groups					
	≤18 years, N=7890		19-25 years, N=7661		26+ years, N=1746	
	N	(%)	N	(%)	N	(%)
**Parents: Other affective disorders**						
Yes	80	(1.0)	66	(0.9)	12	(0.7)
**Parents: Autism**						
Yes	All categories had <5 obs.					
**Parents: ADHD**						
Yes	One of the categories had <5 obs.					
**CYP2D6 phenotype**						
CYP2D6_NM	4884	(61.9)	4802	(62.7)	1084	(62.1)
CYP2D6_IM	2654	(33.6)	2545	(33.2)	582	(33.3)
CYP2D6_PM	352	(4.5)	314	(4.1)	80	(4.6)
**CYP2C19 phenotype**						
CYP2C19_UM	300	(3.8)	302	(3.9)	76	(4.4)
CYP2C19_RM	2071	(26.2)	1947	(25.4)	465	(26.6)
CYP2C19_NM	3470	(44.0)	3341	(43.6)	742	(42.5)
CYP2C19_IM	1867	(23.7)	1921	(25.1)	427	(24.5)
CYP2C19_PM	182	(2.3)	150	(2.0)	36	(2.1)
**Within last 90ds: strong CYP2D6 inhibitor use**						
Yes	72	(0.9)	86	(1.1)	17	(1.0)
**Within last 90ds: moderate CYP2D6 inhibitor use**						
Yes	97	(1.2)	135	(1.8)	43	(2.5)
**Within last 90ds: weak CYP2D6 inhibitor use**						
Yes	One of the categories had <5 obs.					

	Age groups					
	≤18 years, N=7890		19-25 years, N=7661		26+ years, N=1746	
	N	(%)	N	(%)	N	(%)
**Within last 90ds: strong CYP2C19 inhibitor use**						
Yes	182	(2.3)	209	(2.7)	46	(2.6)
**Within last 90ds: moderate CYP2C19 inhibitor use**						
No	7890	(100.0)	7661	(100.0)	1746	(100.0)
**Within last 90ds: weak CYP2C19 inhibitor use**						
Yes	177	(2.2)	186	(2.4)	48	(2.7)
**Within last 90ds: CYP2C19 inducer use**						
Yes	All categories had <5 obs.					
**Within last 90ds: Antiepileptic drug use**						
Yes	64	(0.8)	89	(1.2)	44	(2.5)
**Year as category of first prescription**						
1996-2001	449	(5.7)	17	(0.2)	0	0
2001-2005	2181	(27.6)	1796	(23.4)	0	0
2006-2010	3773	(47.8)	4390	(57.3)	933	(53.4)
2011-2016	1487	(18.8)	1458	(19.0)	813	(46.6)

#Ethnicity was defined based on parental place of birth. Europe means all other countries in Europe except Denmark. If one parent was born outside of Denmark, that region was used. If both parents were born in different regions outside of Denmark, mixed was used.

*For those who had missing information on their own socioeconomic status (SES) we extracted SES from their parents

Abbreviations: UM: ultrarapid metabolizer, RM: rapid metabolizer, NM: normal metabolizer, IM: intermediate metabolizer, PM: poor metabolizer.

For a detailed description of all the variables see supplement table 3.

Table S6. Incidence rates (IR) per 100 person-years with 95% CI of clinical outcomes in individuals using (es)citalopram, sertraline, or fluoxetine and with a depression diagnosis any time between Jan 1, 1995 and Dec 31, 2012, by age groups (≤18, 19-25, >26-36 years).

	**(Es)citalopram**			**Sertraline**			**Fluoxetine**		
	≤18 y.	19-25 y.	26+ y.	≤18 y.	19-25 y.	26+ y.	≤18 y.	19-25 y.	26+ y.
	IR (95% CI)	IR (95% CI)	IR (95% CI)	IR (95% CI)	IR (95% CI)	IR (95% CI)	IR (95% CI)	IR (95% CI)	IR (95% CI)
Switching									
UM	29 (21-41)	32 (24-43)	56 (33-94)	20 (12-33)	23 (12-43)	16 (5-50)			
RM	27 (23-31)	34 (31-38)	43 (35-54)	19 (15-23)	30 (24-38)	37 (26-54)			
**NM**	**28 (25-31)**	**37 (34-40)**	**35 (29-42)**	**16 (14-19)**	**28 (24-34)**	**26 (19-36)**	**17 (14-20)**	**26 (19-35)**	**31 (16-63)**
IM	30 (26-35)	36 (32-40)	34 (26-43)	13 (10-17)	28 (22-35)	32 (22-46)	16 (12-21)	19 (12-31)	20 (5-79)
PM	41 (28-59)	33 (22-49)	40 (19-83)	19 (10-38)	51 (26-97)	18 (3-128)	17 (9-34)	32 (10-100)	41 (6-291)
Discontinuation									
UM	50 (38-65)	80 (66-96)	100 (67-147)	40 (28-56)	81 (58-113)	70 (41-121)			
RM	59 (53-64)	79 (73-85)	92 (79-106)	45 (39-52)	77 (67-88)	103 (83-128)			
**NM**	**54 (50-59)**	**79 (75-84)**	**87 (77-97)**	**39 (36-44)**	**81 (73-89)**	**77 (64-93)**	**47 (42-52)**	**81 (68-96)**	**91 (60-136)**
IM	56 (51-62)	79 (73-85)	82 (70-97)	41 (35-47)	81 (71-92)	78 (62-98)	41 (35-49)	84 (68-105)	69 (33-145)
PM	47 (33-66)	73 (56-95)	91 (56-148)	46 (29-72)	90 (55-147)	36 (9-144)	30 (18-50)	75 (36-158)	82 (20-327)
Emergency room contact									
UM	26 (18-38)	23 (17-32)	28 (14-57)	12 (6-23)	21 (11-43)	23 (8-60)			
RM	23 (20-27)	29 (26-33)	28 (22-36)	16 (12-20)	28 (22-36)	17 (10-29)			
**NM**	**24 (21-26)**	**28 (25-31)**	**25 (20-31)**	**15 (13-18)**	**24 (20-30)**	**21 (14-30)**	**21 (18-25)**	**27 (20-37)**	**31 (15-65)**
IM	23 (20-27)	29 (26-33)	26 (20-34)	17 (13-21)	30 (24-38)	20 (12-31)	23 (18-29)	26 (17-39)	22 (6-88)
PM	29 (18-45)	35 (24-51)	28 (12-67)	13 (5-31)	25 (10-68)	18 (3-128)	16 (8-33)	55 (21-147)	42 (6-300)
Suicide attempt/self- harm									
UM	12 (7-19)	7 (4-13)	9 (3-28)	9 (4-18)	5 (1-18)	0 (0-0)			
RM	11 (9-14)	7 (6-9)	4 (2-8)	9 (6-12)	9 (6-14)	1 (0-9)			
**NM**	**9 (8-11)**	**7 (6-8)**	**3 (2-5)**	**8 (7-10)**	**8 (6-11)**	**1 (0-5)**	**11 (9-14)**	**6 (3-11)**	**8 (2-31)**

IM	12 (9-14)	6 (5-8)	2 (1-5)	10 (7-13)	7 (5-11)	5 (2-13)	15 (11-20)	10 (5-19)	10 (1-70)
PM	23 (15-37)	6 (3-14)	10 (2-39)	17 (8-35)	6 (1-40)	0 (0-0)	9 (3-23)	0 (0-0)*	0 (0-0)*

*0 cases of suicide attempt/self-harm in this group

Abbreviations: UM: ultrarapid metabolizer, RM: rapid metabolizer, NM: normal metabolizer, IM: intermediate metabolizer, PM: poor metabolizer

Table S7. Incidence rate ratios (IRRs) of the association between CYP2C19/CYP2D6 phenotypes and clinical outcomes in individuals using (es)citalopram, sertraline, or fluoxetine and with a depression diagnosis at any time between Jan 1, 1995 and Dec 31, 2012, by age groups (0-18, 19-25, 26-36 y.)

	**(Es)citalopram**			**Sertraline**			**Fluoxetine**		
	≤18 y.	19-25 y.	26+ y.	≤18 y.	19-25 y.	26+ y.	≤18 y.	19-25 y.	26+ y.
	IRR (95%CI) µ	IRR (95%CI) µ	IRR (95%CI) µ	IRR (95%CI) µ	IRR (95%CI) µ	IRR (95%CI) µ	IRR (95%CI) µ	IRR (95%CI) µ	IRR (95%CI) µ
Switching									
UM	0.97 (0.67-1.42)	0.83 (0.61-1.14)	1.69 (0.96-2.97)	1.21 (0.72-2.03)	0.75 (0.38-1.49)	0.60 (0.18-2.01)			
RM	0.94 (0.79-1.13)	0.94 (0.82-1.08)	1.25 (0.93-1.66)	1.14 (0.87-1.49)	1.08 (0.81-1.44)	1.43 (0.86-2.38)			
**NM**	**1**	**1**	**1**	**1***	**1**	**1**	**1**	**1***	**1**
IM	1.03 (0.86-1.23)	0.97 (0.84-1.11)	0.99 (0.72-1.36)	0.78 (0.57-1.06)	1.00 (0.74-1.34)	1.16 (0.70-1.92)	1.00 (0.72-1.39)	0.83 (0.47-1.45)	0.79 (0.12-5.17)
PM	1.64 (1.10-2.43)	0.90 (0.60-1.34)	1.06 (0.49-2.28)	1.20 (0.59-2.46)	2.06 (1.03-4.11)	0.55 (0.07-4.17)	0.87 (0.40-1.88)	1.50 (0.45-5.01)	2.06 (0.17-25.02)
Discontinuation									
UM	0.94 (0.71-1.24)	0.97 (0.80-1.18)	1.27 (0.84-1.93)	1.04 (0.72-1.50)	0.99 (0.69-1.41)	0.88 (0.49-1.59)			
RM	1.07 (0.95-1.21)	1.01 (0.92-1.10)	1.07 (0.88-1.30)	1.15 (0.96-1.37)	0.97 (0.81-1.16)	1.30 (0.96-1.76)			
**NM**	**1**	**1**	**1**	**1**	**1**	**1**	**1**	**1**	**1**
IM	1.05 (0.92-1.20)	1.00 (0.91-1.10)	0.94 (0.77-1.15)	1.01 (0.84-1.21)	1.04 (0.87-1.23)	0.93 (0.69-1.27)	0.87 (0.71-1.07)	1.00 (0.74-1.34)	0.72 (0.25-2.11)

PM	0.89 (0.61-1.30)	0.94 (0.72-1.24)	0.97 (0.58-1.61)	1.20 (0.75-1.91)	1.14 (0.68-1.89)	0.45 (0.11-1.88)	0.58 (0.32-1.03)	0.84 (0.39-1.84)	2.56 (0.41-16.11)
Emergency contact *α*									
UM	1.10 (0.75-1.61)	0.82 (0.59 1.15)	1.38 (0.67-2.88)	0.89 (0.45-1.75)	0.83 (0.40-1.73)	0.62 (0.21-1.88)			
RM	0.95 (0.78-1.14)	1.08 (0.93-1.26)	1.07 (0.76-1.50)	1.02 (0.76-1.38)	1.28 (0.93-1.76)	0.71 (0.36-1.42)			
**NM**	**1**	**1**	**1**	**1**	**1**	**1**	**1**	**1**	**1**
IM	1.00 (0.82-1.22)	1.05 (0.91-1.22)	1.03 (0.72-1.46)	1.06 (0.78-1.45)	1.26 (0.92-1.72)	0.84 (0.45-1.59)	1.02 (0.76-1.37)	1.02 (0.59-1.74)	0.98 (0.12-7.83)
PM	1.43 (0.90-2.29)	1.10 (0.74-1.62)	1.12 (0.45-2.80)	0.77 (0.31-1.89)	1.32 (0.48-3.64)	1.21 (0.16-9.23)	0.61 (0.27-1.39)	3.28 (1.11-9.63)	10.22 (0.35-301.38)
Suicide attempt/self-harm ¥									
UM	1.38 (0.81-2.37)	1.10 (0.62-1.97)	3.30 (0.90-12.04)	0.94 (0.43-2.08)	0.58 (0.14-2.48)				
RM	1.21 (0.92-1.57)	1.08 (0.82-1.43)	1.16 (0.49-2.74)	1.05 (0.71-1.55)	1.07 (0.61-1.87)				
**NM**	**1**	**1**	**1**	**1**	**1**	**1****	**1**	**1**	**1****
IM	1.22 (0.93-1.60)	0.97 (0.72-1.30)	0.68 (0.24-1.99)	1.23 (0.84-1.81)	0.98 (0.55-1.74)		1.19 (0.83-1.73)	1.49 (0.56-3.95)	
PM	2.67 (1.57-4.52)	1.16 (0.51-2.64)	2.93 (0.59-14.62)	1.63 (0.74-3.58)	0.69 (0.09-5.29)		0.93 (0.33-2.59)	0.00 (0.00-0.00)Ψ	

Outcome analyses were done for CYP2C19 UM, RM, NM, IM, or PM if taking sertraline or (es)citalopram and for CYP2D6 NM, IM, or PM if filling a prescription for fluoxetine.

μ -IRR (from Poisson regression analyses) were adjusted for: age, gender, region at index prescription, SES (socio-economic status of parents/adults), number of previous psychiatric diagnoses, co-medication (CYP2C19/CYP2D6 inhibitor or inducer use within the last three months of index date), and calendar year of index prescription;

α -further adjusted for any hospital contact within previous year of index date

¥ -further adjusted for suicide attempt within previous year and antiepileptic drug use (AED) within the last three months of index date

*year was adjusted as a categorical variable

** Adjusted model did not run (due to a small number of cases)

*** Year was adjusted as a continuous variable

Ψ0 cases of suicide attempt/self-harm in this group

Abbreviations: UM: ultrarapid metabolizer, RM: rapid metabolizer, NM: normal metabolizer, IM: intermediate metabolizer, PM: poor metabolizer

Table S8. Numbers for the calculation of the clinical validity and population impact for the statistically significant associations. (presented in table I to IV)

I. **Age: ≤18 years, Drug use: (es)citalopram, CYP2C19: PM, Outcome: Switching**

	**Switching**		
**CYP2C19 phenotype**	Yes	No	Total
PM	28	69	97
Non-PM	778	3164	3942
Total	806	3233	4039

II. **Age: ≤18 years, Drug use: (es)citalopram, CYP2C19: PM, Outcome: Suicide attempt/self-harm**

	**Suicide attempt/self-harm**		
**CYP2C19 phenotype**	Yes	No	Total
PM	18	79	97
Non-PM	341	3601	3942
Total	359	3680	4039

III. **Age: 19-25 years, Drug use: Sertraline, CYP2C19: PM, Outcome: Switching**

	**Switching**		
**CYP2C19 phenotype**	Yes	No	Total
PM	9	21	30
Non-PM	294	1243	1537
Total	303	1264	1567

IV. **Age: 19-25 years, Drug use: Fluoxetine, CYP2D6: PM, Outcome: Emergency contact**: Numbers cannot be report because of <5 cases for at least one of the numbers.
